# The Protective Effect of Auraptene Against Oxidative Stress and Pentylenetetrazol-Induced Chemical Kindling in Mice

**DOI:** 10.22037/ijpr.2019.1100747

**Published:** 2019

**Authors:** Leila Etemad, Mahdieh Zamani, Mehrdad Iranshahi, Ali Roohbakhsh

**Affiliations:** a *Pharmaceutical Research Center, Pharmaceutical Technology Institute, Mashhad University of Medical Sciences, Mashhad, Iran. *; b *Biotechnology Research Center, Pharmaceutical Technology Institute, Mashhad University of Medical Sciences, Mashhad, Iran. *; c *Department of Pharmacodynamics and Toxicology, School of Pharmacy, Mashhad University of Medical Sciences, Mashhad, Iran*.

**Keywords:** Pentylenetetrazol, Auraptene, Chemical kindling, Oxidative stress, Seizure

## Abstract

It is believed that some pitfalls in the treatment of epilepsy such as serious side effects of medications and drug resistance may be resolved by natural compounds. Auraptene belongs to coumarins and is found in citrus peel. We hypothesized that auraptene might have anticonvulsant properties. Kindling was induced by repeated intraperitoneal (IP) injections of pentylenetetrazol (PTZ, 35 mg/kg) with two-day intervals for 24 days in male albino mice. Three groups received IP injections of auraptene (12.5, 25, and 50 mg/kg). Three control groups received vehicle, diazepam (3 mg/kg, IP), and vitamin E (150 mg/kg, IP). Seizure-related behaviors were recorded for 30 min after PTZ injection. Moreover, malondialdehyde and reduced glutathione (GSH) were measured in the brain. The results indicated that auraptene at the dose of 12.5 mg/kg and vitamin E significantly prolonged the latency to stage 2 of seizures (*P *< 0.01). Auraptene at the doses of 25 mg/kg and 50 mg/kg, prolonged the latency to stage 4 (*P *< 0.01) and reduced stage 5 duration of seizures (*P *< 0.01). All doses of auraptene reduced median of seizure scores (*P *< 0.01). The kindled control group had MDA levels similar to intact animals but had a lower concentration of GSH (*P *< 0.001). None of the tested compounds changed the malondialdehyde concentration significantly. However, auraptene at the dose of 50 mg/kg and vitamin E increased GSH levels (*P *< 0.05). The results suggest that auraptene had anticonvulsant effects in PTZ-induced chemical kindling that was mediated by mechanisms other than the antioxidant effect of auraptene.

## Introduction

Epilepsy is a well-known neurological disorder. Recent reports show that 0.5 to 1 percent of people worldwide have epilepsy (1). Epilepsy is recognized by unpredictable and periodic seizures that are induced by abnormal discharges of cerebral neurons. Many people with epilepsy throughout the world lack access to effective treatments. The adverse drug reactions, cost of antiepileptic drugs, the refractory epilepsies, and various forms of epilepsy are among the most important issues facing the health care providers (2). Natural compounds are great sources for finding better antiepileptic treatments. Previous experimental studies show that plants are good sources that may alleviate seizure attacks and comorbid diseases in epileptic patients (3). Accordingly, flavonoids, coumarins, and terpenoids are the main phytochemicals with significant anticonvulsant effects in the preclinical studies (2). Auraptene (7-geranyloxycoumarin) is a well-known and the most abundant prenyloxycoumarin in nature. One of the main sources of auraptene is the plants of the Citrus genus such as grapefruit and orange. Auraptene has significant antioxidant and anti-inflammatory effects (4) and can reduce glutamate in the brain (5). 

Animal models have been used extensively for evaluation of new anticonvulsant drugs. Chemical kindling is a model of epileptic seizures. This model is based on the repeated administration of an initially sub-convulsive dose of a chemical such as pentylenetetrazol (PTZ) (6). This protocol reduces seizure threshold and culminates in a generalized seizure. Drugs with anticonvulsant properties in this model have the potential to be used in the treatment of patients with complex partial epilepsy (7). 

Previous studies demonstrated that during seizure episodes, oxidant/antioxidant balance is perturbed. Numerous studies show that lower antioxidant activity is the main finding during PTZ-induced seizures (8, 9). For example, it was demonstrated that PTZ decreased glutathione peroxidase activity and vitamin E concentration. The study showed that administration of vitamin E not only strengthened the antioxidant capacity but also modulated electroencephalographic recordings following PTZ administration (10). Therefore, we hypothesized that auraptene might be effective in the treatment of epilepsy. Taken together, we aimed to assess the effect of auraptene on PTZ-induced chemical 

kindling.

## Experimental

*Preparation of auraptene*

Auraptene (7-geranyloxycoumarin) was synthesized as described previously (11). In brief, 7-hydroxycoumarin, *trans*-geranyl bromide and DBU (1, 8-diazabicyclo [5.4.0] undec-7-ene) were reacted in acetone at room temperature. Auraptene was purified using column chromatography (petroleum ether/ethyl acetate 9:1 v/v). The structure of auraptene was confirmed by ^1^H- and ^13^C-NMR. Auraptene purity was measured using HPLC as 95%. For injections, auraptene was dissolved in Tween 80, polyethylene glycol 400, and 0.9% saline in 5%, 35%, and 60% v/v order, respectively. 

*Animals*

We used male albino mice weighing 26–35 g. the animals were housed in a room with a 12/12 h light/dark cycle (lights on 07:00 h) and controlled temperature (23 ± 2 °C). The animals had free access to food and water. Each experimental group included ten animals. The animals were randomly divided into different experimental groups to ensure group homogeneity. The method of this study was approved by the Ethics Committee of Mashhad University of Medical Sciences (no. 910972).

*Induction of kindling and experimental design*

For induction of kindling, PTZ (Sigma, India, 35 mg/kg) was injected intraperitoneally (IP) every other day for 24 days (12). Vehicle (10 mL/kg), auraptene (12.5, 25, and 50 mg/kg), vitamin E (Osve, Iran, 150 mg/kg), and diazepam (Chimidaroo, Iran, control drug, 3 mg/kg) were administered intraperitoneally 30 min before PTZ injections. The doses of auraptene and diazepam were selected according to the previous studies (13, 14). Following PTZ injections, each mouse was kept in a Plexiglas box, and its behavior was recorded for 30 min to measure the incidence of convulsions. The intensity of the seizure response was scored on the following scale: 0 = no response; 1 = vibrissae twitching, mouth and facial jerks; 2 = myoclonic body jerks or head nodding; 3 = forelimb clonus; 4 = rearing, falling down, forelimb tonus, and hindlimb clonus; and 5 = tonic extension of hindlimb, status epilepticus (6). The highest response was recorded for each animal for each day. 

The following variables were also recorded: median of seizure stages, stage 2 (S2) and stage 4 (S4) latencies, and stage 5 (S5) duration (15). The means of S2 and S4 latencies and S5 durations for each injection day were calculated and were then averaged over 12 injection days. 

*Biochemical experiments*

*Tissue sampling*

After the last experiment (on day 24) the animals were decapitated and also, their brains were removed, snap-frozen in liquid nitrogen and kept at −80 ºC for biochemical assays.

*Lipid peroxidation assay*

Lipid peroxidation was measured by determination of malondialdehyde (MDA) concentration using UV spectrophotometry. MDA level was expressed as nmol/g tissue (16).

*Reduced glutathione assay*

Brain content of reduced glutathione (GSH) was measured using 2, 2′- dinitro-5, 5′-dithiodibenzoic acid (DTNB) as the reagent. The levels of GSH were measured by UV spectrophotometry at 412 nm and expressed as nmol/g protein (16). 

*Statistical analysis*

The data for seizure stages were expressed as median ± quartiles. The seizure stages were analyzed using Kruskal–Wallis nonparametric one-way analysis of variance followed by two-tailed Mann–Whitney U test. Other data were expressed as mean ± SEM. The comparisons between groups were made by one-way analysis of variance (ANOVA) followed by Dunnett’s test if necessary. *P *< 0.05 was considered significant.

## Results

*The effect of auraptene on PTZ-induced kindling*

**Figure 1:**
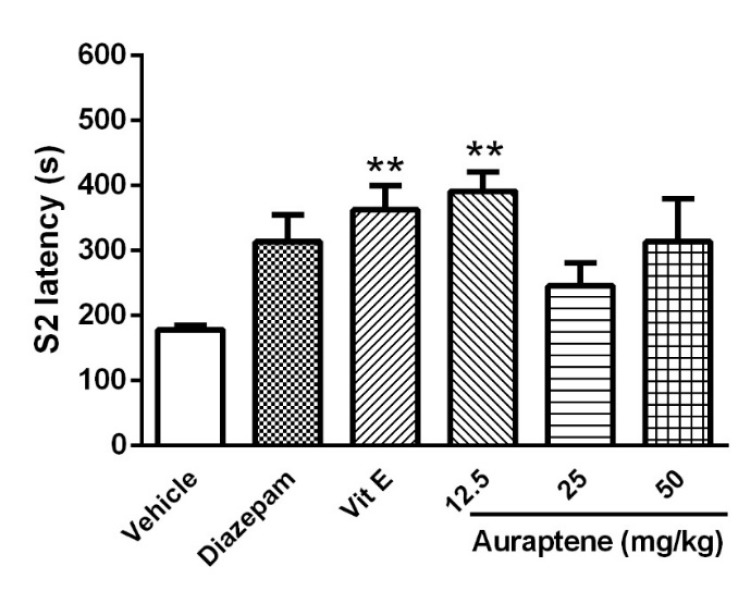
The effect of intraperitoneal injection of auraptene (12.5, 25, 50 mg/kg), vitamin E (150 mg/kg), and diazepam (3 mg/kg) on stage 2 latency in pentylenetetrazol kindled rats. Each bar represents mean ± SEM. In each group n = 10. **: *P* < 0.01 compared with the vehicle group. PTZ: pentylenetetrazol

The results indicated that repeated administration of PTZ for 24 days (vehicle-treated) gradually decreased seizure threshold, which was manifested as increased seizure scores and decreased seizure score latencies. The animals that were pretreated with vitamin E and the low dose of auraptene (12.5 mg/kg) had higher S2L (*P *< 0.01, Figure 1).

**Figure 2:**
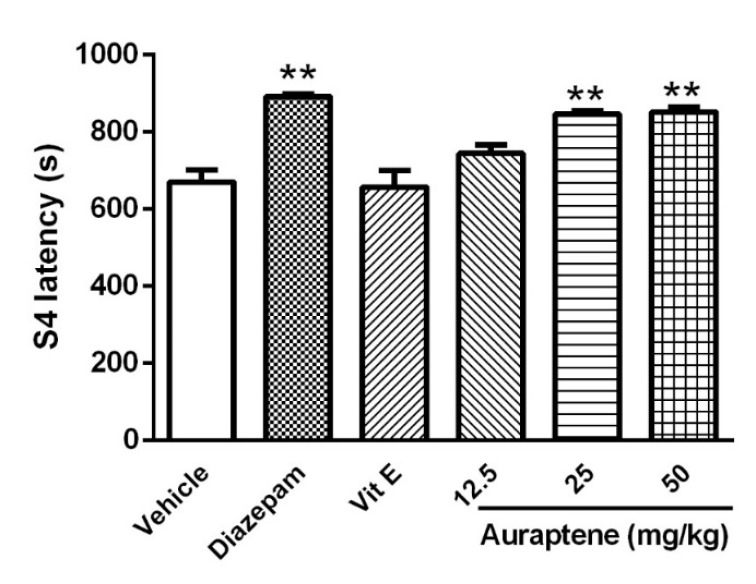
The effect of intraperitoneal injection of auraptene (12.5, 25, 50 mg/kg), vitamin E (150 mg/kg), and diazepam (3 mg/kg) on stage 4 latency in pentylenetetrazol kindled rats. Each bar represents mean ± SEM. In each group n = 10. **: *P *< 0.01 compared with the vehicle group. PTZ: pentylenetetrazol

**Figure 3:**
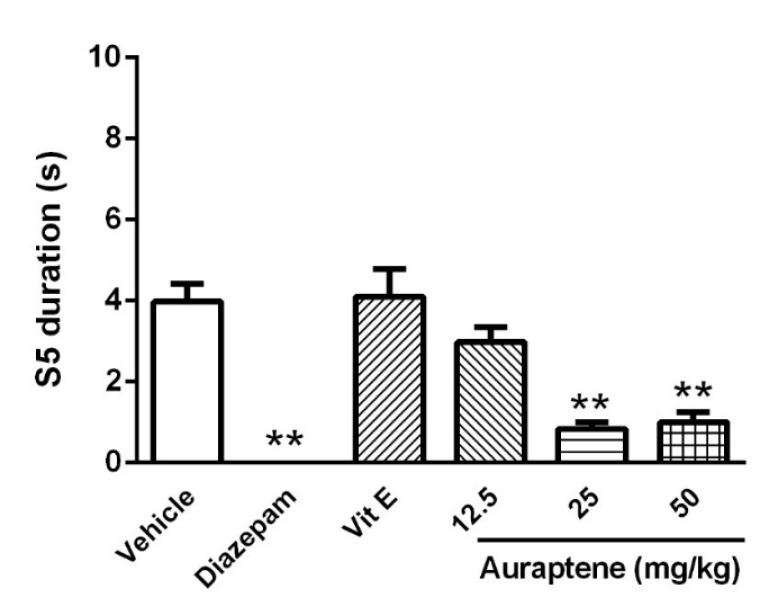
The effect of intraperitoneal injection of auraptene (12.5, 25, 50 mg/kg), vitamin E (150 mg/kg), and diazepam (3 mg/kg) on stage 5 duration in pentylenetetrazol kindled rats. Each bar represents mean ± SEM. In each group n** .10 = : *P *< 0.01 compared with the vehicle group. PTZ: pentylenetetrazol

**Figure 4:**
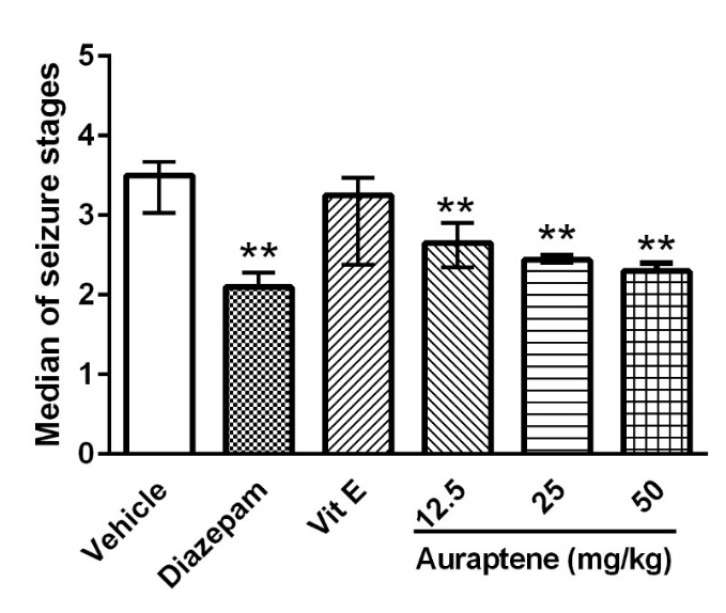
The effect of intraperitoneal injection of auraptene (12.5, 25, 50 mg/kg), vitamin E (150 mg/kg), and diazepam (3 mg/kg) on the median of seizure stages in pentylenetetrazol kindled rats. Each bar represents the median ± quartiles. In each group n = 10. **: *P *< 0.01 compared with the vehicle group. PTZ: pentylenetetrazol

**Figure 5:**
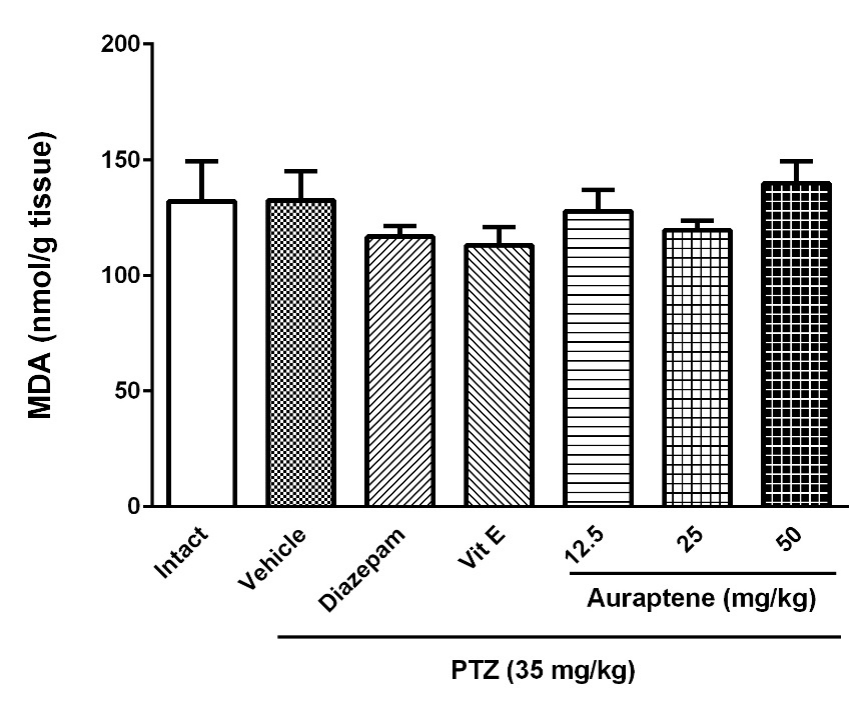
The effect of intraperitoneal injection of auraptene (12.5, 25, 50 mg/kg), vitamin E (150 mg/kg), and diazepam (3 mg/kg) on MDA levels in the brain of pentylenetetrazol kindled rats. Each bar represents mean ± SEM. In each group n 10 =. MDA: malondialdehyde, PTZ: pentylenetetrazol

**Figure 6:**
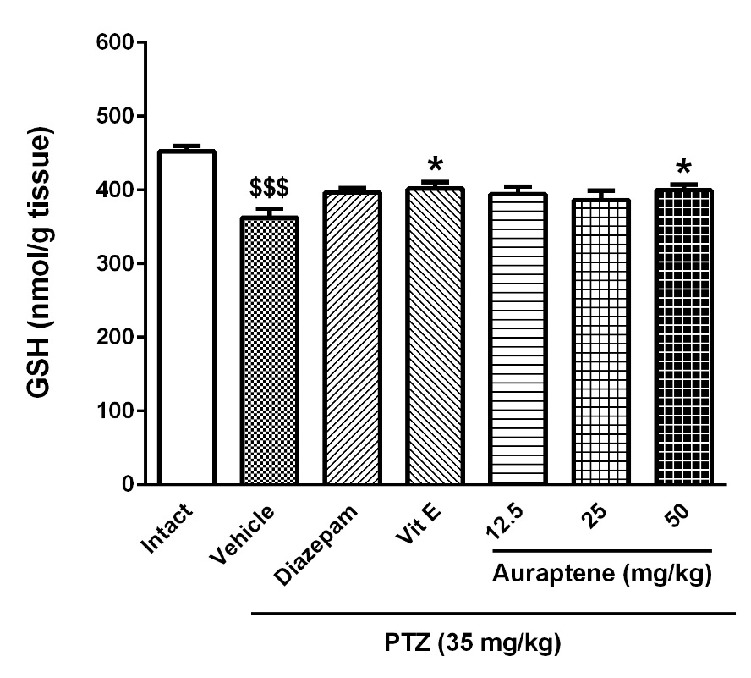
The effect of intraperitoneal injection of auraptene (12.5, 25, 50 mg/kg), vitamin E (150 mg/kg), and diazepam (3 mg/kg) on reduced glutathione (GSH) concentration in the brain of pentylenetetrazol kindled rats. Each bar represents mean ± SEM. In each group n :$$$ .10 = *P *< 0.001 compared with the intact group. *: *P *< 0.05 compared with the vehicle group. PTZ: pentylenetetrazol

However, neither higher doses of auraptene (25 and 50 mg/kg) nor diazepam could change S2L significantly. Auraptene at the doses of 25 and 50 mg/kg increased S4L (*P *< 0.01, Figure 2) and decreased S5D (*P *< 0.01, Figure 3). Similarly, diazepam increased S4L and decreased S5D (*P *< 0.01). S4L and S5D were not different in animals that were pretreated with vitamin E in comparison with the control group.

Comparison of the median score of seizures between different groups showed that auraptene at the doses of 12.5, 25, and 50 mg/kg and diazepam but not vitamin E reduced this parameter significantly (*P *< 0.01, Figure 4). This implies that auraptene induced significant antiepileptic effect in the PTZ-induced kindling model.

*The effect of auraptene on lipid peroxidation and reduced glutathione in the brain*

Figure 5 shows the effect of auraptene on MDA content in the brain. The results show that repeated administration of PTZ did not change the MDA level significantly. Moreover, neither auraptene nor vitamin E changed lipid peroxidation index significantly. The effect of auraptene on reduced glutathione content is presented in Figure 6. The results indicated that repeated administration of PTZ attenuated GSH content in the brain (*P *< 0.001). Administration of auraptene at the dose of 50 mg/kg and vitamin E enhanced reduced glutathione content in the brain (*P *< 0.05). Lower doses of auraptene (12.5 and 25 mg/kg) and diazepam did not change this parameter significantly.

## Discussion

The main finding of the present study is that auraptene had anticonvulsant effect in PTZ kindled mice. It also increased reduced glutathione in the brain similar to vitamin E. However, vitamin E did not produce such anticonvulsant effects in PTZ kindled mice. Compelling evidence from previous studies has shown that some natural compounds have significant anticonvulsant effects (17-19). Coumarins are polyphenolic compounds that possess a diverse range of pharmacological effects. It was reported that administration of PTZ increased free fatty acids, glutathione disulfide, and hydroxyl radicals in various brain regions including the cerebral cortex (8, 20). Also, epileptic patients have been reported to have lower plasma vitamin C content and higher levels of lipid peroxidation in comparison with normal subjects (21). Auraptene, as an herbal compound, was able to decrease main antioxidant enzymes activities including the superoxide dismutase and glutathione peroxidase (9). Therefore, we hypothesized that oxidative stress might have a potential role in PTZ-induced chemical kindling and auraptene would be able to resolve these biochemical changes. The results showed that induction of kindling reduced GSH content in the brain but did not change the MDA level significantly. Similar to these results, Patsoukis *et al*. showed that a single injection of PTZ reduced GSH level in the cerebral cortex of mice but did not affect the MDA levels (8). The present finding is in accordance with previous studies showing that a perturbation in oxidant-antioxidant balance is accounted, at least in part, as a player in the pathogenesis of seizure. Although vitamin E enhanced GSH and decreased stage 2 of seizure attacks, it did not change the median of seizure scores and higher stages of the seizure. Therefore, vitamin E, as a good antioxidant molecule, did not induce a prominent anti-epileptic effect. In other words, vitamin E exhibited a weak anticonvulsant effect. In agreement, a recent study showed that N-acetylcysteine and sulforaphane, which act to increase glutathione, delayed the onset of epilepsy measured at 5 months without modifying the average seizure duration or the incidence of epilepsy in animals (22). Considering the effect of vitamin E and the significant effect of auraptene on GSH at the highest dose, with anticonvulsant effect at lower doses, it may be suggested that the anti-epileptic effect of auraptene was mediated by a mechanism(s) other than modulation of the oxidant-antioxidant system.

It means that longer treatment with antioxidants may induce a better anticonvulsant effect. All these findings show that a combination of antioxidants with current anticonvulsant drugs is an alternative way that would be beneficial for the treatment of epilepsy. 

Previous studies have suggested that seizures originate from two primary brain regions: the forebrain and the brainstem. These studies suggest that seizures characterized by forelimb clonus originate from forebrain structures such as the deep prepiriform cortex or the area tempestas, whereas tonic-clonic seizures are thought to originate from brainstem structures that include the pontine reticular formation and the nucleus reticularis pontis oralis (23, 24). Therefore, it is a possibility that auraptene at the dose of 12.5 mg/kg had more inhibitory effect on the forebrain structures than higher doses and enhanced S2L more than other doses. Due to limited number of studies, the pharmacological effects of auraptene have not been evaluated in detail. Hence, it is hard to suggest the molecular mechanisms behind the anticonvulsant effect of auraptene. Similar to auraptene, there are other coumarin molecules such as esculetin (25) and imperatorin (26) with good anticonvulsant effects. Esculetin has been reported with significant anticonvulsant effect in electroshock-induced convulsions model without sedative and myorelaxant effects. Modulation of voltage-gated and ligand-gated ion channels is a potential mechanism for the anticonvulsant effects of plant compounds. It has been reported that coumarins interact with various voltage-gated ion channels and the benzodiazepine site of the GABAA receptors (27, 28). In accordance, it was reported that the anticonvulsant effect of esculetin was mediated by GABAA receptors. The interaction of coumarin derivatives with GABAA receptors was evaluated by Singhuber *et al*. (29). They showed that these compounds enhance GABA-induced chloride current however, at very high concentrations. Therefore, it is unlikely that this target is the only mechanism that mediates the anticonvulsant effects of coumarins. Auraptene can reduce glutamate concentration as an excitatory neurotransmitter in the CNS (5). This may be another mechanism that explains the anticonvulsant effect of auraptene in the present study. Auraptene has been reported as a ligand for PPARγ as well (30). This nuclear receptor has various physiological effects including modulation of metabolism (31) and inflammation (32). Recent studies show that it is involved in the control of seizure episodes as well. For example, it was demonstrated that single intraperitoneal administration of pioglitazone, as a PPARγ agonist, reduced the proconvulsant effect of PTZ (33, 34). The researchers suggested that PPARγ, through attenuation of proinflammatory cytokines and elevation of nitric oxide synthesis, induced anticonvulsant effects. Therefore, it is a possibility that auraptene induced its anticonvulsant effect through activation of PPARγ. There are reports showing that inflammation has a crucial role in the pathophysiology of epilepsy (35). In agreement, it was demonstrated that the expression of cyclooxygenase-2 (COX-2) was elevated in the pyramidal cells of the hippocampus following kindling. Moreover, following kindling, prostaglandin E2 concentration did not increase in COX-2 knock-out mice despite a significant increase in the wild-type (36). This implies the importance of COX-2 expression during epileptogenesis. Interestingly, there are reports showing that auraptene can change COX-2 expression. For example, auraptene inhibited COX-2 expression in the astrocytes and attenuated microglia activation in the hippocampus (37). Similarly, Okuyama and colleagues showed that auraptene reduced COX-2, interleukin 1β, and TNF-α expressions in the ischemic brain of mice (4). Therefore, another explanation for the anticonvulsant effect of auraptene is decreased expression of COX-2 and other pro-inflammatory chemokines and cytokines. It is worth to mention that IL-1β has a prominent place in the initiation and progression of PTZ-induced epileptogenesis (38, 39).

Compounds with additional neuroprotective, cognition-enhancing, and antiinflammatory activities may be useful in the treatment of epilepsy. In accordance, Epifano *et al*. demonstrated that auraptene produced a significant neuroprotective effect against N-methyl-D-aspartate (NMDA)-induced toxicity in mixed cortical cultures (40). Auraptene has been reported with significant inhibitory action on MAO-B as a neuroprotective mechanism (41). Moreover, auraptene improved reduced ischemia and enhanced memory in a rat model of vascular dementia (14). On the other hand, one of the major pitfalls in the treatment of epilepsy is the interaction of the current antiepileptic drugs with hepatic drug-metabolizing enzymes and therefore with many other drugs. Interestingly, auraptene has been reported as a compound with the lowest interaction with these enzymes (42). Therefore, the neuroprotective properties of auraptene and minimum interaction with hepatic drug-metabolizing enzymes may add benefits to the anticonvulsant effects of this almost safe (43) natural compound. 

In conclusion, auraptene induced significant anticonvulsant effect and increased reduced glutathione. Considering the weak anticonvulsant effect of vitamin E, it was suggested that mechanisms other than the antioxidant effect of auraptene participated in its anticonvulsant effects. 
